# Physicochemical, Biological, and Antibacterial Properties of Four Bioactive Calcium Silicate-Based Cements

**DOI:** 10.3390/pharmaceutics15061701

**Published:** 2023-06-09

**Authors:** Yu-Ji Jang, Yu-Jin Kim, Huong Thu Vu, Jeong-Hui Park, Seong-Jin Shin, Khandmaa Dashnyam, Jonathan C. Knowles, Hae-Hyoung Lee, Soo-Kyung Jun, Mi-Ran Han, Joon-Haeng Lee, Jong-Soo Kim, Jong-Bin Kim, Jung-Hwan Lee, Ji-Sun Shin

**Affiliations:** 1 Department of Pediatric Dentistry, College of Dentistry, Dankook University, 119 Dandaero, Cheonan 31116, Republic of Korea; jyjlove777@gmail.com (Y.-J.J.); miraneee@dankook.ac.kr (M.-R.H.); haeng119@naver.com (J.-H.L.); jskim@dku.edu (J.-S.K.);; 2 Department of Biomaterials Science, College of Dentistry, Dankook University, 119 Dandaero, Cheonan 31116, Republic of Korea; yujin10316426@gmail.com (Y.-J.K.); haelee@dku.edu (H.-H.L.); iris979@hanmail.net (S.-K.J.); 3 Institute of Tissue Regeneration Engineering (ITREN), Dankook University, 119 Dandaero, Cheonan 31116, Republic of Korea; huong.vuthudr@gmail.com (H.T.V.); shurins@naver.com (J.-H.P.); ko2742@naver.com (S.-J.S.); khandmaa@naver.com (K.D.); 4 Department of Nanobiomedical Science & BK21 PLUS NBM Global Research Center for Regenerative Medicine, Dankook University, 119 Dandaero, Cheonan 31116, Republic of Korea; j.knowles@ucl.ac.uk; 5 Drug Research Institute, Mongolian Pharmaceutical University & Monos Group, Ulaanbaatar 14250, Mongolia; 6 UCL Eastman-Korea Dental Medicine Innovation Centre, Dankook University, 119 Dandaero, Cheonan 31116, Republic of Korea; 7 Cell & Matter Institute, Dankook University, 119 Dandaero, Cheonan 31116, Republic of Korea; 8 Division of Biomaterials and Tissue Engineering, Eastman Dental Institute, Royal Free Hospital, Rowland Hill Street, London NW3 2PF, UK; 9 Department of Dental Hygiene, Hanseo University, 46 Hanseo 1ro, Seosan 31962, Republic of Korea; 10 Mechanobiology Dental Medicine Research Center, Cheonan 31116, Republic of Korea

**Keywords:** dental cement, premixed cement, commercial pharmaceutical agent, physicochemical properties, biological properties, antibacterial activity

## Abstract

Calcium silicate-based cement (CSC) is a pharmaceutical agent that is widely used in dentistry. This bioactive material is used for vital pulp treatment due to its excellent biocompatibility, sealing ability, and antibacterial activity. Its drawbacks include a long setting time and poor maneuverability. Hence, the clinical properties of CSC have recently been improved to decrease its setting time. Despite the widespread clinical usage of CSC, there is no research comparing recently developed CSCs. Therefore, the purpose of this study is to compare the physicochemical, biological, and antibacterial properties of four commercial CSCs: two powder–liquid mix types (RetroMTA^®^ [RETM]; Endocem^®^ MTA Zr [ECZR]) and two premixed types (Well-Root™ PT [WRPT]; Endocem^®^ MTA premixed [ECPR]). Each sample was prepared using circular Teflon molds, and tests were conducted after 24 h of setting. The premixed CSCs exhibited a more uniform and less rough surface, higher flowability, and lower film thickness than the powder–liquid mix CSCs. In the pH test, all CSCs showed values between 11.5 and 12.5. In the biological test, cells exposed to ECZR at a concentration of 25% showed greater cell viability, but none of the samples showed a significant difference at low concentration (*p* > 0.05). Alkaline phosphatase staining revealed that cells exposed to ECZR underwent more odontoblast differentiation than the cells exposed to the other materials; however, no significant difference was observed at a concentration of 12.5% (*p* > 0.05). In the antibacterial test, the premixed CSCs showed better results than the powder–liquid mix CSCs, and ECPR yielded the best results, followed by WRPT. In conclusion, the premixed CSCs showed improved physical properties, and of the premixed types, ECPR exhibited the highest antibacterial properties. For biological properties, none of these materials showed significant differences at 12.5% dilution. Therefore, ECPR may be a promising material with high antibacterial activity among the four CSCs, but further investigation is needed for clinical situations.

## 1. Introduction

Calcium silicate-based cement (CSC) is a bioactive cement, similar to mineral trioxide aggregate (MTA) in terms of its composition of calcium and silicate. It has been used to overcome various disadvantages of MTA, such as poor maneuverability, long setting time, and risk of tooth discoloration. Due to its excellent biocompatibility, sealing ability, and antibacterial activity, CSC is widely used in various clinical procedures, such as vital pulp therapy (pulpotomy, pulp capping), apexification, repair of root perforations, root canal filling, and root-end filling [1,2,3]. In recent years, fast-setting products have been released, and there are two types of material: powder–liquid mix type and premixed type. RetroMTA^®^ (RETM; BioMTA, Seoul, Republic of Korea) and Endocem^®^ MTA Zr (ECZR; Maruchi, Wonju, Republic of Korea) are representative powder–liquid mix-type CSCs [4,5].

RETM is composed of a calcium zirconia complex, with biocompatibility similar to that of the initial MTA [6,7]. ECZR is composed of natural pure cement containing zirconium and hardens rapidly due to the presence of small pozzolan particles [8,9].

Premixed CSCs have been introduced recently, and these products offer several advantages over the conventional powder–liquid mix type, including greater uniformity and the ability to inject only the required amount, thus reducing material waste [10,11]. Well-Root™ PT (WRPT; Vericom, Chuncheon, Republic of Korea) and Endocem MTA^®^ premixed (ECPR; Maruchi, Wonju, Republic of Korea) are representative premixed-type CSCs.

WRPT is composed of a calcium aluminosilicate compound containing polypropylene glycol and polyethylene glycol. It is used clinically in the form of a capsule and is known for its excellent antibacterial properties [3,12,13]. ECPR is composed of tricalcium silicate cement, containing dimethyl sulfoxide (DMSO) and propylene methylcellulose, and is delivered in a syringe for direct clinical use. It has shown favorable biocompatibility and chemical properties and was developed to overcome the inconvenience of mixing [7,13,14,15].

There have been several studies on the advantages of RETM in terms of physicochemical, biological, and antibacterial properties, which have addressed some of the limitations of the initial MTA formulation [2,16,17]. However, there is a lack of studies comparing fast-setting powder–liquid CSCs and premixed CSCs. There have been reports that additives may affect the properties of premixed CSCs [18]. Thus, the purpose of this study is to evaluate and compare the physicochemical, biological, and antibacterial properties of four CSCs that are currently widely used clinically. The null hypothesis of this study is that there is no significant difference in physicochemical, biological, and antibacterial properties between powder–liquid mixed CSCs and premixed CSCs.

## 2. Materials and Methods

### 2.1. Sample Preparation

The four commercial materials are described in Table 1. Two kinds of powder–liquid mix-type CSCs (RetroMTA^®^ [RETM] and Endocem^®^ MTA Zr [ECZR]) and two kinds of premixed-type CSCs (Well-Root™PT [WRPT] and Endocem MTA^®^ premixed [ECPR]) were used. The CSCs underwent a hydration reaction with water or water-containing liquids. Of the powder–liquid mix types, RETM was mixed with the provided liquid at a W/P ratio of 3 drops to 0.3 g, and ECZR was mixed with sterile distilled water (DW) at a W/P ratio of 0.14 cm^3^ to 300 mg. For the premixed types, since no instructions were provided by the manufacturers regarding the amount of liquid to use, samples were prepared to be the same size as ECZR, and experiments were conducted using 0.14 cm^3^ sterile DW, which was the same amount used for ECZR. According to the manufacturers’ instructions, each sample (inner diameter: 20.0 mm; height: 2.0 mm) was prepared using circular Teflon molds for tests. The powder–liquid mix-type CSCs were mixed and placed in the molds, and the premixed-type CSCs were immediately squeezed and applied to the molds. The molds with the samples were kept in wet conditions at 37 °C for 24 h to achieve complete setting.

### 2.2. Physicochemical Analysis

The flow and film thickness were measured according to the International Standard Organization (ISO) 6876:2012 protocol.

Surface characterization, crystal structure analysis, elemental analysis, ion release analysis, and pH measurements were performed using circular Teflon molds.

#### 2.2.1. Flow

To evaluate the flowability, 0.05 mL of each material was placed on a plate (40 mm width, 40 mm length, 5 mm height), creating a circle. Within 60 s of the start of mixing, a second glass plate was placed on top of the CSC, and additional mass was added to a total of (120 ± 2) g. Then, the weight was removed 10 min after the start of mixing, and the maximum and minimum diameters of the compressed material were measured using a digital caliper (Mitutoyo, Mitutoyo Corp., Kawasaki, Japan). This test was repeated 3 times for each material.

#### 2.2.2. Film Thickness

To evaluate the film thickness, the thickness of the two glass plates (15 mm width, 15 mm length, and 5 mm height) combined was accurately measured with a digital micrometer (Absolute Digimatic 500-197, Mitutoyo Corp, Kawasaki, Japan). Within 60 s of the start of mixing, 0.01 g of the mixed sample was placed in the center of the glass plate, a second glass plate was placed in the center of the sample, and a load of 150 N was applied perpendicularly to the glass plate. At 10 min after mixing, the thickness of the two glass plates and the sample was measured with a micrometer. The film thickness of the CSC was calculated as the difference in thickness between the samples with and without the glass plates, and the average value was obtained after performing the test 3 times per experimental group.

#### 2.2.3. Surface Analysis

After setting, the samples were dried and coated with gold–palladium (20/80). The surface morphology of the samples was analyzed using scanning electron microscopy (SEM, Sigma 500, ZEISS, Oberkochen, Germany). All analyses were carried out with an accelerating voltage of 15 kV. Images were obtained with a magnification of 1000×. The surface roughness was measured at three different spots on each sample using a surface roughness tester (SJ-400, Mitutoyo, Kawasaki, Japan, *n* = 3).

#### 2.2.4. Crystal Structure Analysis

To assess the crystal structure of the samples, X-ray diffraction (XRD, Rigaku, Ultima IV, Tokyo, Japan) was conducted with Cu Kα radiation at 40 mA and 40 kV over a 2θ range from 5° to 80° with a sampling width of 0.02° and a scan speed of 4 °/min. The XRD was controlled using Jade version 6.1 software (MDI, Livermore, CA, USA).

#### 2.2.5. Elemental Analysis

Energy-dispersive spectroscopy (EDS; Noran System Seven, Thermo Fisher Scientific, Waltham, MA, USA) was used to detect the elemental compositions on each specimen surface after the samples were prepared for SEM. The accelerating voltage was set at 15 kV, and the X-ray intensity was set at 100 counts per second. The proportions of elements were calculated as weight %.

#### 2.2.6. Ion Release

After setting, each CSC sample was eluted in 5 mL of phosphate-buffered saline (PBS) at 37 °C for 24 h according to ISO 10993-5. The released liquid was filtered through a 0.2 µm filter, and 3 mL of the filtered liquid was transferred to a test tube that contained 30 µL of concentrated nitric acid (HNO_3_; Wako Pure Chemical Industries, Osaka, Japan). Lithium (Li), strontium (Sr), calcium (Ca), magnesium (Mg), aluminum (Al), iron (Fe), silica (Si), sulfur (S), and phosphorus (P) ions were chosen and measured using inductively coupled plasma-atomic emission spectrometry (ICP–AES; OPTIMA 8300, PerkinElmer, Boston, MA, USA). All samples were examined three times, and the results were averaged. The ion release concentration of each element was reported in ppm (=mg/L).

#### 2.2.7. pH Measurements

After setting, each CSC sample was eluted in 5 mL of PBS at 37 °C, and the pH was measured using a Thermo Orion Star pH meter (Thermo Scientific Inc., Melbourne, Australia) at 3, 5, 12, 24, 72, and 168 h after immersion. Before the pH test, the pH meter was calibrated using standard solutions at pH 4.01, 7, and 10.01. The PBS (5 mL) was exchanged for each measurement. Each measurement was repeated three times, and the results were averaged.

### 2.3. Biological Analysis

#### 2.3.1. Primary Culture of Stem Cells from Human Exfoliated Deciduous Teeth (SHED)

SHED were extracted for clinical purposes from an exfoliated deciduous tooth (obtained from a 6-year-old male) at Dankook University’s Dental Hospital, following guidelines approved by the ethical committee of the Dankook University Dental Hospital’s institutional review board (IRB number DKUDH 2019-10-001). All participants signed an informed consent form. The extracted tooth was placed in 5 mL of Hanks’ balanced salt solution (HBSS, Welgene, Daegu, Republic of Korea) containing 1% penicillin–streptomycin (PS, Gibco, Thermo Fisher, Waltham, MA, USA) at 4 °C. The pulp tissue was chopped and submerged in a 2 mg/mL collagenase type I solution (Worthington Biochemical, Lakewood, NJ, USA). The dissociated cell suspension was suspended in 1 mL of growth medium (αMEM, MEM Alpha Modification, with Ribo- and deoxyribonucleosides, with L-glutamine, HyClone, Logan, UT, USA) containing 15% fetal bovine serum (FBS, Corning, NY, USA), 2 mM L-glutamine (Thermo Fisher), and 0.1 mM L-ascorbic acid phosphate (Thermo Fisher) and centrifuged at 1500 rpm for 5 min at room temperature. Following the removal of the supernatant, 1 mL of growth medium was added to the cell pellet, and this single-cell solution was filtered using a 75 µm trainer (Falcon, Corning, NY, USA) before being plated on culture dishes (60 mm × 15 mm, Falcon, Corning) and incubated at 37 °C in a 5% CO_2_ atmosphere with 95% humidity. The culture medium was changed after 48 h of incubation and then every 7 days thereafter. Single-cell-derived colonies were collected and subcultured after 10 to 14 days. The cells were then digested with 0.25% EDTA-trypsin (Thermo Fisher) and passaged when the cell confluence was 80–90%. For the following experiments, cells from passages 4 to 10 were used. Cell viability tests using the Cell Counting Kit-8 (CCK-8, Dojindo Laboratories, Kumamoto, Japan) assay and phalloidin and 4′,6-diamidine-2′-phenylindole dihydrochloride (DAPI) staining, as well as cell differentiation tests using the alkaline phosphatase (ALP) assay, were performed using the SHED.

#### 2.3.2. Cell Viability Test

A cell viability test was conducted on SHED using the CCK-8 assay and staining with phalloidin and DAPI. A total of 100 µL of 5000 cells/mL were grown for 24 h at 37 °C in a humidified atmosphere of 5% CO_2_ in each well of two 96-well plates (SPL Life Sciences, Pocheon, Gyeonggi-do, Republic of Korea) using supplemented media. After washing with PBS (100 µL), the cells were cultured for another 24 h with 100 µL of serially diluted extract and positive control (0%). After being rinsed with PBS (100 µL), the cells were cultivated for another 24 h with 100 µL of serially diluted extract and positive control (0%). The final extract percentages in the culture medium were 50%, 25%, 12.5%, and 6.25%, with supplemented medium serving as a positive control (0%). The CCK-8 assay was used to detect cell cytotoxicity. Each well received ten microliters of CCK-8 solution, and the 96-well plates were placed in a CO_2_ incubator for two hours to allow the reaction to occur. The optical density (OD) of each well was then determined by means of a microplate absorbance reader (VarioskanTM LUX, Thermo Fisher Scientific) at 450 nm to determine the cell viability. The CCK-8 assay is based on measuring the dehydrogenase activity of metabolically active living cells capable of converting a slightly yellow tetrazolium salt (WST-8) into orange-colored WST-8 formazan. All analyses were carried out in triplicate and independently. The cell viability was calculated as follows:

(1) $$\mathrm{cell}\mathrm{viability}\left(\%\right)=\frac{OD(experiment)-OD(blank)}{OD(control)-OD(blank)}\times 100$$

After the CCK-8 assay confirmed the cell viability, the cells were rinsed with PBS, fixed with 4% paraformaldehyde (PFA), and permeabilized with 0.2% Triton X-100. Rhodamine phalloidin was applied and incubated for 25 min before adding DAPI to the wells and incubating for 5 min. A fluorescence microscope (IX71, Olympus, Tokyo, Japan) was used to capture cell images.

#### 2.3.3. Odontoblastic Differentiation Test

To determine the effects of the CSCs on odontoblastic differentiation, cells were cultured with the tested materials in odontoblastic medium (OM) for 7 days. SHED at a density of 5000 cells/well were seeded on a 48-well plate overnight, and then the medium was changed to odontoblastic differentiation medium (αMEM, 10% FBS, 1% PS, 10 mM transforming growth factor-β1) with different concentrations of material extract dilutions (3.125%, 6.25%, 12.5%, 25%) in 50 µM to 200 µM OM with 100 nM dexamethasone (Sigma) and 50 µg/mL ascorbic acid at 37 °C in 5% CO_2_. Differentiated SHED without material extract were used as a positive control. The medium was refreshed every 3 days for 1 week. ALP was measured to determine the effectiveness of differentiation. On Day 7, ALP staining was performed with a staining kit (SIGMAFASTTM BCIP^®^/NBT tablet, Sigma-Aldrich, St. Louis, MO, USA). On Day 7, differentiated cells were fixed with 4% paraformaldehyde (Tech & Innovation) at 37 °C for 15 min before being stained with ALP solution dissolved in DW for 1 h in the dark at 37 °C. Light microscopy (Olympus IX71, Shinjuku, Tokyo, Japan) was used to obtain optical images to measure the ALP activity after staining and washing five times with distilled water. Quantification was performed using ImageJ software Java 8 (Bethesda, MD, USA). The extracts were prepared according to ISO 10093-5:2009 [19].

### 2.4. Antibacterial Activity

*Enterococcus faecalis* (*E. faecalis*, ATCC 19433) was purchased from the American Type Culture Collection (ATCC) in Manassas, VA, USA. Bacterial colonies were streaked onto plates containing brain heart infusion agar (BHIA; BD Difco, Franklin Lakes, NJ, USA) and incubated at 35 ± 2 °C for 24 h. A single colony was then transferred to brain heart infusion broth (BHIB; BD Difco, Franklin Lakes, NJ, USA) and incubated with shaking.

To obtain sample extracts, CSC specimens were eluted in DW for 24 h. Mixtures were prepared through combining 1 mL of *E. faecalis* at a concentration of 2 × 10^6^ colony forming units (CFUs)/mL with 1 mL of the sample extracts (100%) eluted in a 12-well plate. The final bacterial concentration was 10^6^ CFUs/mL, and the concentration of the sample extracts was 50%. The mixtures were shaken for 1 h using a digital shaker, and then 200 µL of PrestoBlue (Molecular Probes, Eugene, OR, USA) was added. The mixtures in the 12-well plates were transferred to a 98-well plate at one-hour intervals, with 100 µL per well. The 98-well plate was observed under a light microscope, and the OD was measured at 570–600 nm using a microplate reader (BioTek, Winooski, VT, USA) (*n* = 5). The plate counting method was used on agar medium to observe live bacteria more accurately at 3 h.

### 2.5. Statistical Analysis

All data from repeated tests were expressed as the mean ± standard deviation. The Statistical Package for Social Sciences software version 21.0 (SPSS Inc., Chicago, IL, USA) was used to analyze the significant differences among the CSC groups using one-way analysis of variance (ANOVA) and Tukey’s honest significant difference (HSD) test. A *p* value of less than 0.05 was considered to indicate statistical significance.

## 3. Results

### 3.1. Physicochemical Properties

#### 3.1.1. Flow and Film Thickness

The flow and film thickness are summarized in Table 2. In the flow test, the powder–liquid mix type CSCs, RETM and ECZR, showed lower flow values (7.06 ± 0.55 mm and 7.03 ± 0.27 mm, respectively). On the other hand, WRPT and ECPR, which are premixed types, showed higher values (13.83 ± 0.35 mm and 20.18 ± 1.16 mm, respectively). Significant differences (*p* < 0.05) were observed among RETM, WRPT, and ECPR and among ECZR, WRPT, and ECPR. In the film thickness test, of the powder–liquid mix type CSCs, RETM had a thickness of 233.33 ± 47.25 µm, and ECZR had a thickness of 246.66 ± 41.63 µm. On the other hand, WRPT and ECPR, which are premixed types, showed lower values of 143.00 ± 13.00 µm and 30.00 ± 5.00 µm, respectively. Significant differences (*p* < 0.05) were observed among RETM, WRPT, and ECPR and among ECZR, WRPT, and ECPR. As seen in these results, the higher the flowability, the lower the film thickness.

#### 3.1.2. Surface Analysis

**Figure 1:**
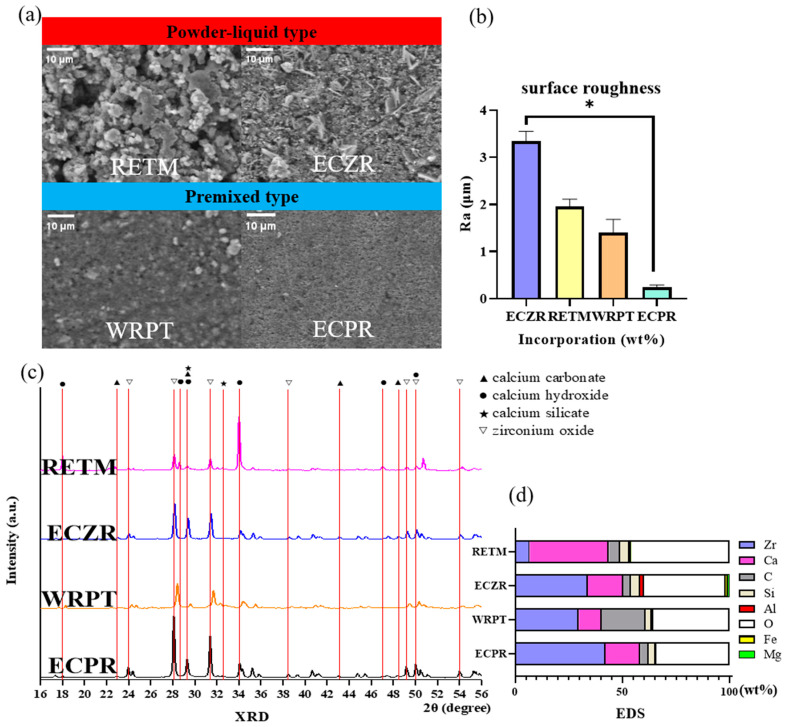
Schematic representation of the surface analysis conducted using SEM, crystal structure analysis conducted using XRD, and elemental analysis conducted using EDS for the four CSCs. (**a**) SEM images of each material after 24 h of immersion in PBS buffer at 37 °C. The top row shows powder–liquid type CSC surface images at 1000× magnification, while the bottom row shows premixed type CSCs at 1000× magnification. The premixed types (WRPT and ECPR) exhibited a more uniform surface structure than the powder–liquid mix types (RETM and ECZR). (**b**) The surface roughness results show that the powder–liquid mix types (RETM and ECZR) were rougher than the premixed types (WRPT and ECPR). (**c**) XRD patterns of each material after 24 h of immersion in PBS buffer at 37 °C. The ECZR and ECPR patterns exhibited peaks for CaCO_3_ at 22.94°, 29.28°, 42.99°, 46.99°, and 48.35°. The RETM pattern showed a high-intensity calcium hydroxide peak at 33.99°. (**d**) EDS analysis of the composition of each material after 24 h of immersion in PBS buffer at 37 °C. WRPT had a higher C content (20.54%) than the other materials. ECPR had a higher Zr content (42.18%) than the other materials. RETM had a higher Ca content (36.93%) than the other materials. ECZR was the only cement that contained Fe and Mg. The error bars indicate standard deviations. The asterisks indicate significant differences (*p* < 0.05). Zr = zirconium, Ca = calcium, C = carbon, Si = silica, Al = aluminum, O = oxygen, Fe = iron, and Mg = magnesium. RETM, RetroMTA^®^; ECZR, Endocem^®^ MTA Zr; WRPT, Well-RootTM PT; ECPR, Endocem^®^ MTA premixed.

The surfaces of the hydrated CSCs are presented in Figure 1a. The premixed types (WRPT and ECPR) exhibited a more uniform surface than the powder–liquid mix types (RETM and ECZR). The surface roughness results are presented in Figure 1b. The powder–liquid mix types (RETM and ECZR) were rougher than the premixed types (WRPT and ECPR).

#### 3.1.3. Crystal Structure

Figure 1b displays the XRD patterns of the four CSCs. All four materials consisted mostly of zirconium oxide crystals. Zirconium oxide peaks were noted at 23.94°, 28.06°, 31.36°, 34.05°, 35.20°, 49.15°, and 50.01°. Calcium hydroxide peaks were noted at 17.99°, 18.31°, 33.99°, 47.02°, and 50.72°. Calcium silicate peaks were noted at 29.26° and 32.28°. ECZR and ECPR exhibited peaks for calcium carbonate (CaCO_3_) at 22.94°, 29.28°, 42.99°, 46.99°, and 48.35°.

#### 3.1.4. Elemental Analysis

Table 3 and Figure 1d display the elemental composition of each material. All the cements were found to contain Ca, Si, carbon (C), zirconium (Zr), Al, and oxygen (O). ECZR was the only cement that contained Fe and Mg. WRPT had a higher C content (20.54%) than the other materials. ECPR had a higher Zr content (42.18%) than the other materials. RETM had a higher Ca content (36.93%) than the other materials.

#### 3.1.5. Ion Release

**Figure 2:**
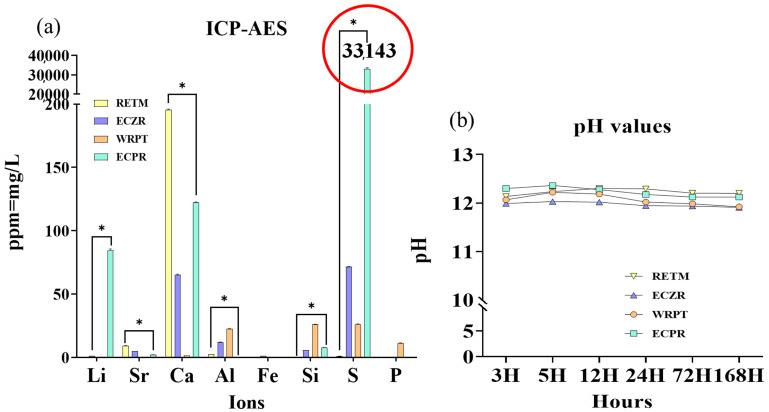
Schematic representation of ICP–AES and pH analyses of four CSCs. (**a**) Li, Sr, Ca, Mg, Al, Fe, Si, S, and P ions were selected. The highest S and Li ion releases were observed for ECPR (*p* < 0.05). The red circle indicates that 300 times more S ions were detected in ECPR than in the other materials. The highest Ca and Sr ions were observed for RETM (*p* < 0.05). Additionally, WRPT showed higher releases of Si, Al, and P ions (*p* < 0.05), and ECZR showed Fe ion release (*p* < 0.05). (**b**) pH changes for the four CSCs after 3, 5, 12, 24, 72, and 168 h of contact with PBS. Before the pH test, the pH meter was calibrated using standard solutions at pH 4.01, 7, and 10.01. The PBS (5 mL) was exchanged for each measurement. All four materials had a high alkaline pH of approximately 12. ECZR consistently showed the lowest pH value compared to the other groups at all time points (3, 5, 12, 24, 72, and 168 h). The asterisks indicate significant differences (*p* < 0.05).

The means, standard deviations, and statistical comparisons for ion release (mg/L) are presented in Table 4 and Figure 2a. All CSCs exhibited ion release of Ca, Al, Si, and S. RETM, ECZR, and ECPR also released Sr ions, while ECZR and ECPR released Li ions, and ECZR released Fe ions. WRPT released P ions. The highest S and Li ion releases were observed for ECPR (*p* < 0.05). The highest Ca and Sr ion releases were observed for RETM (*p* < 0.05). Additionally, WRPT showed higher releases of Si, Al, and P ions (*p* < 0.05), and ECZR showed Fe ion release (*p* < 0.05).

#### 3.1.6. pH

The pH values are shown in Figure 2b. The results indicated that all four materials had highly alkaline pH values of approximately 12. ECZR consistently showed the lowest pH value compared to the other groups at all time points (3, 5, 12, 24, 72, and 168 h).

### 3.2. Biological Properties

#### 3.2.1. Cell Viability

**Figure 3:**
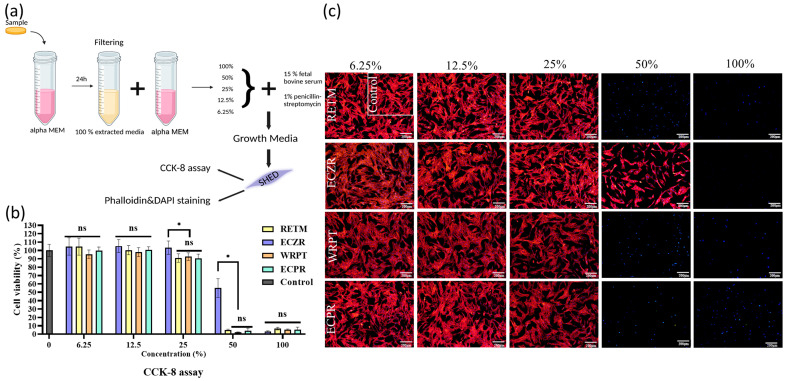
Schematic representation of the initial cell viability of SHED after exposure to four CSCs. To evaluate the cell viability after exposure to the CSCs at different dilution ratios (50%, 25%, 12.5%, and 6.25%), SHED were exposed to CSCs at different dilution ratios, and then viability assays (CCK-8 assay and phalloidin and DAPI staining) were performed. (**a**) Schematic representation of the experiment: samples were immersed in culture medium (αMEM) at 37 °C in a humidified atmosphere of 5% CO_2_. After 24 h, the medium was extracted and diluted at different concentrations (6.25%, 12.5%, 25%, 50%, and 100%) for the viability test. (**b**) The CCK-8 assay revealed that the cell viability after exposure to ECZR increased markedly to approximately 60%, whereas cell viability after exposure to the other materials showed no change at a concentration of 50%. Although the cell viability increased by approximately 90% for all four materials when the concentration of the extract was reduced to 25%, the cell viability after exposure to ECZR was slightly higher than those after exposure to the other materials (*p* < 0.05). At lower concentrations (6.25% and 12.5%), there was no significant difference among the four materials in terms of the resulting cell viability. (**c**) Representative images of phalloidin- and DAPI-stained SHED at different concentrations; the most significant difference among the samples was seen at a concentration of 50% material extraction, and the number of intact cells was much higher for ECZR than for the other materials. The error bars indicate standard deviations. The asterisks indicate significant differences (*p* < 0.05). Represents ns = nonsignificant, scale bar = 200 µm.

Figure 3 illustrates the cell viability results of the extracts of four types of cement. The cell viability was evaluated through measuring cytotoxicity against SHED using the CCK-8 assay and was later reevaluated through confocal microscopy images to confirm the nucleus and cell morphology via staining with phalloidin and DAPI. The 100% extract exhibited severe cytotoxicity, resulting in less than 10% cell viability. However, when the concentration of the extract was reduced by 50%, the cell viability after exposure to ECZR increased markedly to approximately 60%, whereas the cell viability after exposure to the other materials showed no change. Although the cell viability increased by approximately 90% for all four materials when the concentration of the extract was reduced to 25%, the cell viability after exposure to ECZR was slightly higher than that after exposure to the other materials (*p* < 0.05). At lower concentrations (6.25% and 12.5%), there was no significant difference in the cell viability after exposure to the four materials. To confirm the cell viability under culture conditions, confocal microscopy was used. The cell images confirmed the results of the cell viability assay through showing only live ECZR cells in the 50% extract group compared to the control group. All the images of the 12.5% and 6.25% extracts showed cell viability similar to that of the control (Figure 3c). As expected, extract dilution in the medium decreased the cytotoxicity of the CSCs.

#### 3.2.2. Odontoblastic Differentiation

**Figure 4:**
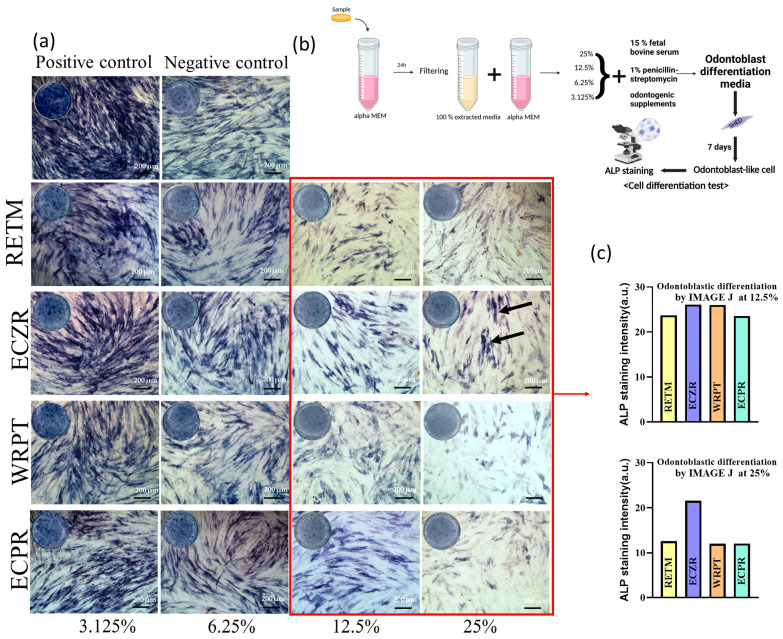
Schematic of experiments and results of odontoblastic differentiated SHED exposed to diluted CSCs. To determine the effects of the CSCs on odontoblastic differentiation, cells were cultured with the tested materials in odontoblastic differentiation medium for 7 days. Differentiated SHED without material extract were used as a positive control. (**a**) ALP staining revealed that cells exposed to ECZR showed better odontoblastic differentiation capacity than cells exposed to the other tested materials. The black arrows indicate higher ALP staining in the Zr group with 25% dilution. All four materials showed similar ALP staining at 12.5%, 6.25%, and 3.125%. (**b**) Schematic representation of the experiment: samples were immersed in culture medium (αMEM) at 37 °C in a humidified atmosphere of 5% CO_2_. After 24 h, the medium was extracted and diluted to different concentrations (3.125, 6.25, 12.5, 25) for odontoblastic differentiated assays. SHED were cultured in odontoblastic differentiation medium with different concentrations of CSCs for 7 days, and the culture medium was refreshed every 3 days. ALP staining was performed using a staining kit. (**c**) ImageJ analysis for quantifying cell differentiation at 12.5% and 25% dilutions. The amount of differentiation in the red square was quantified and displayed as a graph. ECZR resulted in a higher degree of cell differentiation at a 25% dilution. However, there was no difference in the material at 12.5% dilution. scale bar = 200 µm.

The odontoblastic potential of four CSCs was analyzed in vitro (Figure 4). The 3.125%, 6.25%, 12.5%, and 25% extracts were subjected to ALP activity examinations. Since there was severe cytotoxicity in the 50% and 100% extracts, the above concentrations were excluded from the cell differentiation experiments. More differentiation was observed after exposure to ECZR at a 25% extract concentration than after exposure to the other 3 materials (Figure 4a). All four materials showed similar ALP staining at 12.5%, 6.25%, and 3.125% concentrations (Figure 4a,c).

### 3.3. Antibacterial Activity

**Figure 5:**
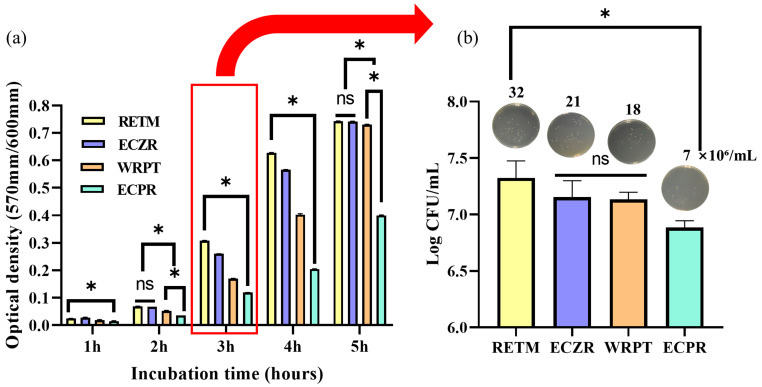
Schematic representation of antibacterial effects using four CSCs analyzed with *E. faecalis*, 10^6^ CFUs/mL. (**a**) The optical density of *E. faecalis* using PrestoBlue. *E. faecalis* cultured with ECPR exhibited the highest antibacterial activity relative to that observed in the presence of the other materials. (**b**) The colony formation units (CFU/log10) after 3 h of contact were also evaluated using a microplate reader. The number of colonies was 7, 18, 21, and 32 for ECPR, WRPT, ECZR, and RETM, respectively. The antibacterial activity was the highest for ECPR and the lowest for RETM. The error bars indicate standard deviations. The asterisks indicate significant differences (*p* < 0.05). Represents ns = nonsignificant.

The effect of CSCs on the viability of *E. faecalis* was tested in vitro (Figure 5). The microbial OD was measured at each incubation time. The antibacterial activity was significantly different for each material after 3 h of incubation time. ECPR exhibited the highest antibacterial activity, followed by WRPT, ECZR, and RETM (Figure 5a, *p* < 0.05). The colony formation units (CFU/log10) after 3 h of contact environment were also evaluated using a microplate reader (Figure 5b). The number of colonies was 7, 18, 21, and 32 for ECPR, WRPT, ECZR, and RETM, respectively. The highest antibacterial activity was observed for ECPR, followed by WRPT, ECZR, and RETM. A significant difference was observed between ECPR and RETM.

## 4. Discussion

Recently, several commercial CSCs with significantly reduced initial setting times under 10 min have been developed. This improvement is beneficial for one-visit treatments, where the restoration can be placed immediately after applying the CSCs. In contrast, the previous CSC product, ProRoot, had a long setting time of 279 min, which posed a significant disadvantage [8]. Premixed CSCs, with their ease of use and operability, are favored as pharmaceutical agents in clinical treatments. No study has investigated the physical, biological, and antibacterial properties of premixed CSCs. We evaluated the physicochemical, biological, and antibacterial properties of four fast-setting CSCs, including premixed types. The initial setting times of the products were determined to be 123.33 ± 5.77 s for ECZR, 146.67 ± 5.77 s for RETM, 260 ± 17.32 s for ECPR, and 460 ± 17.32 s for WRPT [20].

Physical properties, such as film thickness and flow, strongly affect the material’s clinical performance, thus affecting their sealing ability [4]. In this study, premixed-type CSCs showed higher flow and lower film thicknesses than powder–liquid-type CSCs (*p* < 0.05). According to a previous study, additional components, such as polyethylene glycol in WPRT and hydroxypropyl methylcellulose in ECPR, of premixed CSCs could increase the flowability [21]. This could contribute to better adaptation to various irregularities present in the root canal system and improve the material’s ability to seep into the canal or perforation areas [18,22]. According to ISO 6876 [23] standards for root canal sealers, a film thickness of less than 50 μm and a flowability of more than 17 mm are needed. Only ECPR satisfies these conditions. This indicates that ECPR can be considered a root canal sealer as well as a pulp capping material. 

SEM analysis is a commonly used technique for the observation of the surface of materials, along with EDS for element analysis and XRD for crystallization [24,25]. During surface observation, the premixed type exhibited a more uniform and less rough surface. Material uniformity can reduce the influence of operator skill [11].

In the XRD observations, all four materials were found to be composed mainly of zirconium oxide crystals. In the past, MTA traditionally used bismuth oxide as a radiopacifier, which resulted in a delay in the setting reaction of CSCs [26,27]. In contrast, the use of zirconium oxide in these materials did not hinder the setting reaction [28,29]. CaCO_3_ was observed in ECZR and ECPR. CaCO_3_ acts as a nucleation site for calcium silicate hydrate, affecting the induction period of the setting [25].

In the EDS observations, all four materials included Zr, Ca, O, C, Si, and Al. The ECZR was observed to include Mg and Fe. WRPT had a higher C content than the other materials. Identification of C on the surfaces suggested the presence of a polymer, such as polyethylene glycol, which is not reported in the Material Safety Data Sheet (MSDS) data from the manufacturer [30].

ICP–AES results were obtained based on the EDS results from this study and the composition information in the MSDS provided by the manufacturer. Ion release is affected by the nature of the mineral particles as well as the network structure of the cement, which is responsible for water sorption, solubility, and the material’s permeability to water diffusion (i.e., porosity) [31]. The highest release of calcium ions was observed for RETM, which is composed of a calcium zirconia complex. The release of calcium ions from materials is important for the differentiation of pulp cells and maintaining dentin mineralization and the formation of a dentin bridge through the enhancement of pyrophosphatase activity. Therefore, the ability to release calcium ions is a key factor for successful pulp therapy due to the role of calcium in pulp cell differentiation and hard tissue mineralization [31,32,33].

The highest release of sulfide ions was observed for ECPR, which is composed of calcium sulfate and DMSO. This is consistent with the results of a previous study [14]. DMSO has a high dielectric constant and the ability to solvate polymers and adhesives due to its low surface energy; it is an aprotic solvent that possesses the necessary polarity to disrupt the self-associative tendencies of water and form stable complexes with it and can help improve dentin wettability [34,35]. DMSO has intrinsic antimicrobial properties and has been reported to have antibacterial effects against Gram-positive bacteria [15]. Thus, the release of high concentrations of S ions could have an antibacterial effect on *E. faecalis* in our study [14,36].

The highest release of Al ions was observed in WRPT, a cement composed of a calcium aluminosilicate compound synthesized by the manufacturer, which may affect initial cell growth and cause cytotoxicity [37]. Additionally, Al increases the permeability of bacterial membranes, which decreases their ability to function [38]. However, Al is also known to cause tooth discoloration, which is a common issue with CSCs used in dental procedures [39].

The highest release of Li ions was observed for ECPR due to the presence of lithium carbonate, which can help reduce the setting time of the material through promoting the hydration of calcium aluminate. Additionally, the release of Li ions can increase the pH of the material, resulting in a more alkaline environment [35,40].

P ions were observed in WRPT. Calcium hydroxide can also react with P ions to form amorphous calcium phosphate, which can then be converted into hydroxyapatite. Hydroxyapatite exhibits excellent biocompatibility, which manifests in the form of minimal tissue toxicity and foreign-body reaction, as well as osteoinductivity and osteogenicity.

Mg and Fe were observed only in ECZR. Mg has been reported to play a crucial role in human bone growth and repair through stimulating osteoblast proliferation, with its most important function being the regulation of calcium transport. Nevertheless, metal oxides such as ferric oxide have also been linked to discoloration [41,42].

The effect of the above ions on cell viability and differentiation and their antibacterial properties were not investigated in this study, so additional research is needed. It has been reported that the initial pH of CSC mixed with water is known to be approximately 10.2, and after 3 h, a strong alkalinity of approximately pH 12.5 has been observed due to a reaction with Ca ions [18]. Ca ions are produced in high quantities from calcium hydroxide and through the decomposition of calcium silicate hydroxide, which leads to an alkaline pH [43]. Strong alkalinity is believed to be crucial in biological activity, to be related to the antibacterial effect against *E. faecalis* and to have mineralization effects [18,44,45], as shown by previous studies [46,47,48]. McHugh [49] stated that a high alkaline pH of more than 11.5 is bactericidal to *E. faecalis*. All materials used in this study satisfied this condition. One disadvantage of this alkaline pH is possible high cytotoxicity. However, this initial cytotoxicity could also be considered an advantage [50]. All the CSCs in this study had a pH value of approximately 12, with RETM showing the highest Ca ion concentration and pH value. The high pH of WRPT was maintained despite its low Ca ion concentration, which is dependent not only on the released calcium ion concentration but also on the composition of the cement and the reaction of Ca ions already combined with other ions to form compounds or crystal structures, which may have influenced the hydroxyl group and helped to maintain a high pH. Notably, a high Ca ion concentration does not always indicate a high pH [51]. For example, ECZR always had a lower pH than the other materials due to its composition of pozzolanic cement, as the consumption of calcium hydroxide during the pozzolanic reaction reduced the amount of free calcium hydroxide produced in the hydration reaction [29].

In the CCK-8 assay results, all four materials showed a cell viability rate of 7% or less at a concentration of 100%. The highly alkaline CSCs might deleteriously affect surrounding pulpal cells in a concentration of 100% in the cytotoxicity test [52]. At a 50% dilution, only ECZR recovered the initial cell viability. A trial conducted by Yun J et al. [5] concluded that SHED cultured in ECZR showed more cell viability than those cultured in RETM. This was consistent with our study [53]. At a 25% dilution, the cell viability in all samples recovered to nearly the control levels (Figure 3b). There was a significant difference between ECZR and the other 3 materials in both the 25% and 50% cytotoxicity tests (*p* < 0.05). According to ISO 10993-5 [19], a reduction in cell viability by more than 30% is considered a cytotoxic effect. At a dilution of 12.5%, all the CSCs showed a high cell viability of more than 90%. This result suggests that the four materials showed similar biocompatibility at 12.5% or less. As expected, the cytotoxicity of the CSCs was reduced at low concentrations.

When the CSCs are in direct contact with cells, they are cytotoxic, which can be explained by the high pH during setting [54,55]. A high pH compromises the integrity of the cytoplasmic membrane through disrupting organic components (proteins and phospholipids) and interfering with nutrient transport [56]. As a result, cell viability may be reduced. Under clinical conditions, however, the extracts from the material are diluted by the surrounding tissue fluids. Consequently, the pH of the material extracts contacting the cells may be lower in a clinical setting than in in vitro studies [2].

ALP activity is an indicator of the successful differentiation of preodontoblasts into odontoblasts and the formation of new bone [57]. ALP releases free phosphate ions, which react with calcium ions to form a calcium phosphate precipitate, the molecular unit of hydroxyapatite. A previous study reported that ALP exhibited pronounced expression one week after its induction as an early marker of osteogenic/odontoblastic differentiation [57]. We evaluated the effect of the studied materials on odontoblast differentiation and mineralization via ALP staining on Day 7. In our results, ALP staining revealed that the cells exposed to ECZR showed better odontoblast differentiation capacity than the cells exposed to the other tested materials at 25% dilution. However, all four materials showed similar cell differentiation at a low 12.5% dilution. Therapeutic ions, such as Ca, Si, Sr, and Mg, are known to stimulate osteoblast differentiation. ECZR had a relatively low pH and was the only material containing Mg ions, which have been reported to play an important role in human bone growth and repair through stimulating osteoblast proliferation and regulating calcium transport [41]. These results may explain the high cell differentiation observed with ECZR. However, according to Lee et al., the mineralization potential using Alizarin Red S staining of ECZR was lower than that of RETM. In Lee’s study, ECZR showed higher pH and lower viability than RETM [58]. This may be a result of the different methods. Unfortunately, the reasons why different CSCs give rise to different biological responses have not been fully investigated in this study. Additional research is needed.

Bacterial infection following endodontic treatment remains a significant complication. Choosing a material with high antibacterial activity helps to decrease or limit the growth of any remaining bacteria, even after treatment in infected defects [59]. *E. faecalis* is a Gram-positive bacterium and is a representative bacterium found in failed endodontic treatment. In particular, it has been found to have a significant correlation with pain history and radiographic periapical lesions of primary teeth. Thus, it has been employed widely for testing the antibacterial activity of endodontic materials [60,61]. Extracts from all the CSCs had a high pH between 11.5 and 12.5 and showed antibacterial activity, as measured via OD measurements of *E. faecalis*. This is consistent with a previous study showing that the growth of *E. faecalis* was inhibited at pH values higher than 11.5 [62]. The effect of pH on antibacterial activity was mainly due to Ca(OH)_2_ releasing hydroxyl ions in an aqueous environment. The cellular components of the bacteria were affected by hydroxyl ions, which function as free radicals with the highest level of reactivity with other biomolecules [63].

However, contrary to the theory that high pH indicates antibacterial activity, RETM, which had the highest pH, showed the lowest antibacterial activity in this study. On the other hand, ECPR inhibited the growth of *E. faecalis* most effectively (*p* < 0.05), despite having a similar pH. All four materials showed high pH but had significantly different antibacterial activities, as determined via OD measurements. Moreover, there was a significant difference in antibacterial activity between ECPR and RETM (*p* < 0.05), as shown in the CFU/log10 counting method (Figure 5b). The difference in antibacterial activity among the materials in this study was likely affected by the structure and composition of the materials. It has been reported that the inclusion of additives in hydraulic cement can affect its biocompatibility and, in particular, antibacterial reactivity [64,65]. Previous studies have shown that antibacterial properties can be affected not only by Ca or hydroxyl ions but also by S and Al ions [36,66]. ECPR, which is composed of calcium sulfate and DMSO, had the highest S ion content. In the fields of medicine, pharmacy, chemistry, and synthetic biology, DMSO is recommended as an organic solvent for drug development due to its intrinsic antimicrobial properties. It has also been reported to have antibacterial effects against Gram-positive bacteria such as *Mycobacteroides abscessus* [14,15,36]. Therefore, the release of high concentrations of S ions from ECPR could have a significant antibacterial effect. WRPT had the highest Al ion content, which increased the permeability of bacterial membranes and damaged the bacteria [38]. In addition, it has been reported that polyethylene glycol in WRPT exhibits antibacterial properties against early acid-producing bacteria and shows excellent bacterial resistance through preventing bacterial adhesion through improved hydrophilicity resulting from increased surface energy and surface roughness [67,68]. ECZR, on the other hand, had the second-highest S ion content and the second-highest Al ion content. Differences in these components may have additional effects on antimicrobial properties beyond the effect of pH. Based on these results, the proposed null hypothesis was rejected.

Finally, the results suggested that there is a complex relationship between the cytocompatibility and antibacterial activity of CSCs and between pH and Ca ion release. Unfortunately, the mechanisms of influence of these factors were not investigated in this study. Although more study is needed, these findings are innovative in that they apply to premixed products that are used clinically but have rarely been studied and, thus, may provide guidelines for optimizing the properties of CSCs in further studies.

There are limitations to this study. First, since this experiment used only SHED and *E. faecalis*, the results may differ when other stem cells and bacteria are used. Second, since the experimental materials in this study were tested in vitro in a set state, there may be differences in the actual oral environment. Third, the reasons for which structures or elements cause differences in biological responses have not been fully investigated in this study. Further study on the relationship between cytotoxicity and antibacterial activity related to the chemical components or microstructure of CSCs is necessary. An evaluation of the concentration of additive components is required to balance cytocompatibility. Nevertheless, our findings may offer valuable guidelines for optimizing the properties of CSCs for use in clinical practice.

## 5. Conclusions

The premixed-type CSCs exhibited a more uniform and less rough surface, higher flowability, and lower film thickness than the powder–liquid mix CSCs. ECZR and ECPR, which exhibited fast setting times among the powder–liquid mix and premixed types, respectively, had a CaCO_3_ structure. All four CSCs had a pH value of approximately 12, with RETM showing the highest Ca ion concentration and pH value. ECZR showed a lower pH value and cytotoxicity with higher cell differentiation at 25% dilution. However, all the materials showed similar levels of cell viability and cell differentiation at low concentrations. In the assessment of antibacterial activity, ECPR showed the highest antibacterial activity. ECPR may be a promising material in that it has improved maneuverability and antibacterial properties and shows cytocompatibility at 12.5% dilution comparable to that of powder–liquid type CSCs. However, further investigation is needed to understand the mechanisms through which variations in concentrations of specific components impact biological properties or their effects in clinical situations.

## Figures and Tables

**Table 1:** Manufacturer and composition of the materials used in the present study.

Category	Material	Abbreviation	Composition	Manufacturer	Lot Number
Powder–liquid mix type	RetroMTA^®^	RETM	Calcium zirconia complex, silicon dioxide, aluminum oxide, calcium carbonate, zirconium oxide	BioMTA, Seoul, Republic of Korea	RM1305D01
	Endocem^®^MTA Zr	ECZR	Natural pure cement (silicon dioxide, aluminum oxide, calcium oxide, ferric oxide, magnesium oxide), zirconium dioxide	Maruchi, Wonju, Republic of Korea	LB2103230323
Premixed type	Well-Root^TM^ PT	WRPT	Calciumaluminosilicate compound, filler, thickening agent, zirconium oxide	Vericom, Chuncheon, Republic of Korea	WT010100
	Endocem^®^ MTA premixed	ECPR	Tricalcium silicate, calcium aluminate, calcium sulfate, dimethyl sulfoxide, thickening agents (lithium carbonate, hydroxypropyl methylcellulose, phyllosilicate mineral), zirconium dioxide	Maruchi, Wonju, Republic of Korea	NWSEG110530

**Table 2:** The results of flow and film thickness (mean ± SD).

Material	Film Thickness (µm)	Flow (mm)
RETM	233.33 ± 47.25 ^c^	7.06 ± 0.55 ^a^
ECZR	246.66 ± 41.63 ^c^	7.03 ± 0.27 ^a^
WRPT	143.00 ± 13.00 ^b^	13.83 ± 0.35 ^b^
ECPR	30.00 ± 5.00 ^a^	20.18 ± 1.16 ^c^

Within each column, the significant differences between groups are indicated by different superscript letters.

**Table 3:** Elemental composition determined using EDS.

Material	Zr	Ca	C	Si	Al	O	Fe	Mg
RETM	6.82 ± 0.60	36.93 ± 0.76	5.26 ± 0.21	4.16 ± 0.09	1.01 ± 0.02	45.82 ± 0.15	-	-
ECZR	33.90 ± 1.39	16.45 ± 0.35	3.86 ± 0.09	4.02 ± 0.11	1.85 ± 0.10	37.91 ± 0.97	1.30 ± 0.08	0.72 ± 0.10
WRPT	29.72 ± 3.16	10.62 ± 2.26	20.54 ± 3.52	2.69 ± 0.45	0.84 ± 0.19	35.58 ± 1.95	-	-
ECPR	42.18 ± 1.33	16.28 ± 0.36	3.89 ± 0.49	2.98 ± 0.29	0.40 ± 0.04	34.28 ± 0.43	-	-

The elemental composition data are presented as the mean ± SD (wt%).

**Table 4:** Concentrations of ions released from the samples (ppm = mg/L) (mean ± SD).

Material	Li	Sr	Ca	Al	Fe	Si	S	P
RETM	-	9.40 ± 0.06 ^c^	195.75 ± 0.33 ^d^	2.60 ± 0.02 ^b^	-	0.46 ± 0.00 ^a^	0.93 ± 0.15 ^a^	-
ECZR	1.12 ± 0.01 ^a^	5.02 ± 0.03 ^b^	65.17 ± 0.54 ^b^	12.21 ±0.07 ^c^	1.26 ± 0.01	5.84 ± 0.01 ^b^	71.61 ± 0.15 ^c^	-
WRPT	-	-	1.43 ± 0.01 ^a^	22.76 ± 0.17 ^d^	-	26.02 ± 0.16 ^d^	26.23 ± 0.27 ^b^	11.55 ± 0.04
ECPR	84.69 ± 0.98 ^b^	2.20 ± 0.02 ^a^	122.26 ± 0.42 ^c^	0.56 ± 0.00 ^a^	-	7.71 ± 0.10 ^c^	33143.95 ± 544.80 ^d^	-

The ion release data are shown as the mean ± SD. Within each column, the significant differences between groups are indicated by different superscripted letters.

## Data Availability

Not applicable.
